# Antenatal diagnosis of congenital hepatic hemangioma: a case report

**DOI:** 10.4076/1757-1626-2-6829

**Published:** 2009-08-07

**Authors:** Sofia Vargas Cabrita, Sónia Gonçalves, Henrique Rodrigues, Nuno Guerra, Paulo Moura

**Affiliations:** 1 Obstetrics Department of University Hospital of CoimbraRua Miguel Torga, 3030-165, CoimbraPortugal; 2 Radiology Department of University Hospital of CoimbraPraceta Prof. Mota Pinto 3000-075 CoimbraPortugal

## Abstract

Hepatic tumors in children account for only 1 to 5% of all pediatric tumors. Hepatic hemangioma is, however, the third most common tumor of the liver in childhood. We report a case of an antenatal diagnosis of a hepatic tumor detected on a first obstetric ultrasound, at 26^th^ week of gestation. It revealed a complex, predominantly solid hepatic lesion with 3 × 3 cm and a marked, essentially peripheral, Doppler blood flow. Fetal echocardiography showed a normal heart besides a vena cava displacement by the hepatic mass. Fetal Hepatic hemangioma was suspected. Follow-up ultrasounds were unchanged. Pregnancy evolved well. At 36 weeks of gestation was spontaneously delivered a 3300 g boy whose examination revealed a visible thoracoabdominal circulation and a palpable liver. No skin lesions, namely hemangiomas or petechiae were identified. Postnatal magnetic resonance imaging confirmed the diagnosis of Hepatic hemangioma. Treatment was initiated with prednisolone followed by interferon. After 2 years, there is no active lesion.

## Introduction

Hepatic tumors in children account for only 1 to 5% of all pediatric tumors. Hepatic Hemangioma (HH) is, however, the third most common tumor of the liver in childhood [1-3]. It is a proliferative endothelial cell lesion with characteristic initial rapid growth followed by frequent spontaneous involution. Replacing the old and controversial nomenclature (“hepatic hemangioendothelioma”) emerged the latter classification of vascular anomalies, as proposed by Mulliken and Glowacki and by the International Society for the Study of Vascular Anomalies (ISSVA) [3,4]. Two different types of hemangiomas that may affect the liver in children were introduced: infantile hemangiomas and congenital hemangiomas. The latter may be Rapidly Involuting congenital hemangioma (RICH) or Noninvoluting congenital hemangioma (NICH).

While small HH can be asymptomatic lesions incidentally discovered during imaging of the abdomen, larger tumors may act as arteriovenous shunts resulting in high-output congestive heart failure and hepatic dysfunction that may lead to fetal non-immune hydrops (unrelated to Rh disease). Other complications include coagulopathy, hemolytic anemia and tumor rupture. Diagnosis is most often based on characteristic radiological findings (obstetric and postnatal ultrasound, computed tomography and magnetic resonance imaging) [5-7]. Biopsy should be used in selected cases as it may result in massive haemorrhage. Natural history of small, asymptomatic HH is spontaneous involution and no treatment is required. Larger or symptomatic lesions need aggressive management which may include medical therapy with corticosteroids (CS) or agents with strong antiangiogenic effect (interferon alpha, cyclophosphamide, vincristine or actinomycin D), irradiation, selective embolization or surgical intervention (vessel ligations, tumor excision or hepatic transplantation) [8-10].

## Case presentation

A 27 year-old, healthy, gesta 3 para 2 Caucasian Portuguese woman presented for an obstetric ultrasound at 26 weeks of gestational age (GA) to an outpatient sonography department. Until then she had no pregnancy surveillance. A complex, predominantly solid hepatic lesion was noted, measuring approximately 3 cm in diameter. No other fetal anatomic abnormalities were detected.

**Figure 1.**
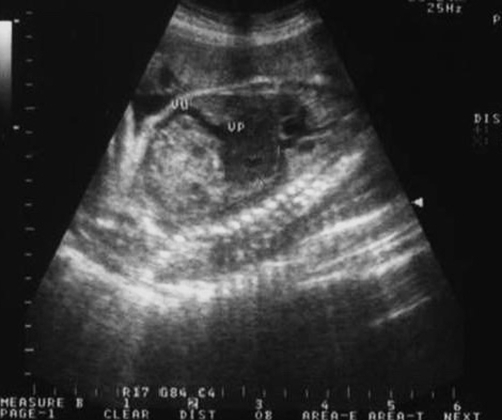
Hepatic lesion at prenatal ultrasound.

The patient was referred to our centre and a follow-up ultrasound at 30^th^ gestational week revealed a heterogeneous, predominantly solid hepatic lesion with mostly hypoechoic texture, a diameter of 3 cm and a marked, essentially peripheral, Doppler blood flow (Figure 1). Fetal Hepatic hemangioma was suspected.

Fetal echocardiography showed a normal heart with vena cava displacement by the hepatic mass. Obstetric ultrasound repeated at 33 weeks GA showed similar characteristics of the hepatic formation, with an average fetal growth and a normal umbilical artery Doppler.

Gestational Diabetes was diagnosed at 34 weeks GA, with no need for insulinotherapy. There was no other pregnancy-related complication.

Follow-up ultrasound at 35^th^ gestational week was unchanged. At 36 weeks of gestation was spontaneously delivered a 3300 g boy with an Apgar score of 9/10 at 1^st^ and 5^th^ minutes respectively.

Newborn physical examination revealed a visible thoracoabdominal circulation, abdominal distension and a palpable liver. No skin lesions, namely hemangiomas or petechiae were identified.

**Figure 2.**
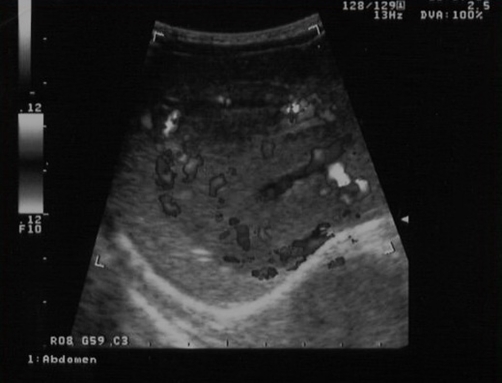
Hepatic lesion at postnatal ultrasound; marked, peripheral Doppler blood flow.

Besides an abnormal gamma-glutamyltransferase, laboratory data was unremarkable (Table 1). Chest and abdominal radiography were normal. Postnatal abdominal ultrasound revealed a 5 cm heterogeneous lesion occupying essentially the hepatic caudate lobe, showing marked hepatopetal blood flow (Figure 2).

**Table 1.** Laboratory findings

	Newborn	5^th^ day	4^th^ month	6^th^ month	2 years
Hemoglobin (g/dL)	14.6	13.7	12.3	12.1	11.7
Platelets (/mm^3^)	211,000	172,000	189,000	223,000	215,000
Gamma Glutamyl Transpeptidase (U/L)	682	930	1325	315	90
Alkaline Phosphatase (U/L)	93	438	523	278	153
Lactate dehydrogenase (U/L)	192	238	427	191	159
Aspartate aminotransferase (U/L)	8	216	312	174	29
Alanine aminotrasferase (U/L)	6	117	207	132	18
Total/Conjugated Bilirubin (mg/dL)	1.5/0.9	4.3/3.1	3.7/2.3	1/<0.5	<1/<0.5
Alpha-fetoprotein (ng/mL)	-	18,600	10,430	358	1.3

**Figure 3.**
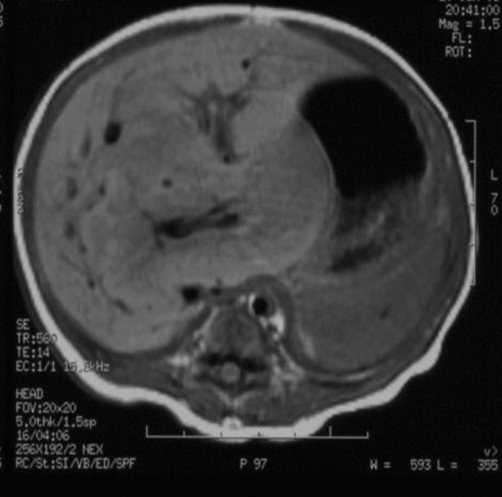
Lesion surrounding the hilum, similar in signal with hepatic parenquima.

**Figure 4.**
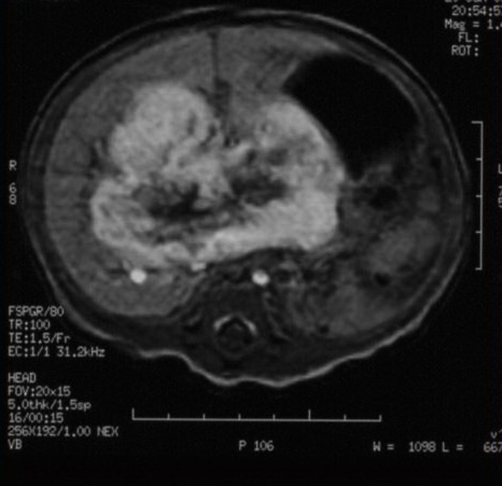
Hyperintense hepatic lesion in T2- weighted images.

**Figure 5.**
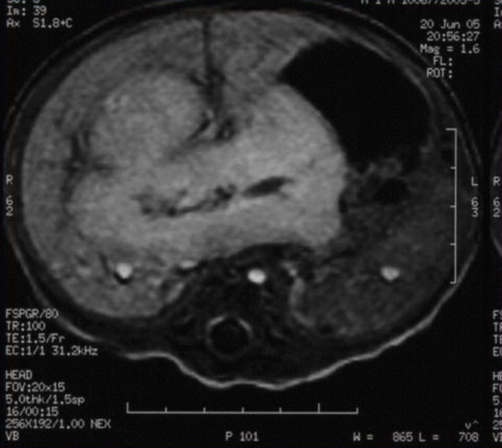
Homogeneous late phase enhancement.

Unenhanced magnetic resonance imaging (MRI) showed a nodular mass with 7.3×5.1 cm affecting both lobes of the liver with involvement of celiac trunk and hepatic hilum, marked by low signal intensity on T1-weighted images and high signal intensity on T2-weighted images (Figures 3,4). After intravenous contrast administration the lesion revealed peripheral enhancement with centripetal filling (slow wash-out) (Figure 5). Because of the increased vascular supply, the tumor was associated with a striking decrease in the aortic calibre distal to the celiac artery origin. A deterioration of the liver function tests (LFTs) was verified, with a considerable increase in liver and cholestatic enzymes (Table 1).

Treatment was initiated with prednisolone (3 mg/Kg/day) but after 4 months the lesion showed the same characteristics, with a sustained deterioration of LFTs. An alternative medical treatment with subcutaneous α-2a-interferon was then started; beginning with 500.000 U/m^2^/day with daily dose increased up to 1 MU/m^2^/day for 3 months, with progressive regression of the hepatic tumor and LFTs.

Besides clinical surveillance, LFTs and MRI were used in follow-up. After 2 years the boy is asymptomatic, with normal LFTs (Table 1) and with last MRI control showing a nodular hepatic lesion between left and middle hepatic artery, measuring 1.5×0.9×0.5 cm suggesting residual hemangioma.

## Discussion

Hepatic Hemangioma is a rare benign neoplasia representing, however, the most common vascular tumor of the liver in the neonate [1,2]. As proposed by Mulliken and Glowacki and according to the International Society for the Study of Vascular Anomalies (ISSVA) [3,4], hemangiomas that may affect the liver in children can be classified as: infantile hemangiomas and congenital hemangiomas. When large enough, this tumor can act as an arterio-venous shunt and cause serious consequences *in utero* and post-natally. Some case reports of antenatal ultrasound diagnosis have been described showing the enormous value of a previous evaluation by allowing the pediatric team to be prepared.

Since biopsy may result in massive haemorrhage, diagnosis is most often based on characteristic radiological findings [5-7]. A postnatal evaluation of the tumor, namely with MRI, is essential to decide what is the best treatment strategy (expectant, medical, surgical) as this is considered the technique of choice for imaging of hepatic vascular lesions [8].

In our case, a complex fetal hepatic lesion was diagnosed at an obstetric ultrasound performed at 26 weeks GA. Differential diagnosis included benign and malignant conditions such as congenital hemangioma, mesenchymal hamartoma, hepatoblastoma and embryonal sarcoma. The strongest alternative diagnosis for this large, solitary, stable lesion was hemangioma. In fact, the stabilized growth of the lesion with no associated anomalies allowed a more confident surveillance of the pregnancy. Neonatologist team was aware of the case so that postnatal abdominal ultrasound and MRI were promptly provided. As MRI was consistent with HH, biopsy was avoided.

Natural history of small, asymptomatic HH is spontaneous involution and no treatment is required. Larger or symptomatic lesions need aggressive management which may include medical therapy with CS or agents with strong antiangiogenic effect, irradiation, selective embolization or surgical intervention [8-10].

As it was a large, symptomatic tumor with a deterioration of the hepatic function, expectant management was excluded and medical treatment with CS was initiated. This is usually the first choice as it is associated with fewer side effects. The unresponsiveness to CS determined the use of a second-line medical therapy with interferon, with very good results as seen in literature [10]. Given the description of rare cases of malignization, long term monitoring should be maintained at least until complete resolution.

## Abbreviations

CS: Corticosteroids
GA: Gestational age
HH: Hepatic hemangioma
LFTs: Liver function tests
MRI: Magnetic resonance imaging
